# Elimination of Falciparum Malaria and Emergence of Severe Dengue: An Independent or Interdependent Phenomenon?

**DOI:** 10.3389/fmicb.2018.01120

**Published:** 2018-05-30

**Authors:** Ib C. Bygbjerg, Lone Simonsen, Karin L. Schiøler

**Affiliations:** ^1^Department of Public Health, University of Copenhagen, Copenhagen, Denmark; ^2^Department of Science and Environment, Roskilde University, Roskilde, Denmark

**Keywords:** dengue, falciparum malaria, co-morbidity, cross-protection, heterologous immunity, interaction

## Abstract

The global malaria burden, including falciparum malaria, has been reduced by 50% since 2000, though less so in Sub-Saharan Africa. Regional malaria elimination campaigns beginning in the 1940s, up-scaled in the 1950s, succeeded in the 1970s in eliminating malaria from Europe, North America, the Caribbean (except Haiti), and parts of Asia and South- and Central America. Dengue has grown dramatically throughout the pantropical regions since the 1950s, first in Southeast Asia in the form of large-scale epidemics including severe dengue, though mostly sparing Sub-Saharan Africa. Globally, the WHO estimates 50 million dengue infections every year, while others estimate almost 400 million infections, including 100 million clinical cases. Curiously, despite wide geographic overlap between malaria and dengue-endemic areas, published reports of co-infections have been scarce until recently. Superimposed acute dengue infection might be expected to result in more severe combined disease because both pathogens can induce shock and hemorrhage. However, a recent review found no reports on more severe morbidity or higher mortality associated with co-infections. Cases of severe dual infections have almost exclusively been reported from South America, and predominantly in persons infected by *Plasmodium vivax*. We hypothesize that malaria infection may partially protect against dengue – in particular falciparum malaria against severe dengue – and that this inter-species cross-protection may explain the near absence of severe dengue from the Sub-Saharan region and parts of South Asia until recently. We speculate that malaria infection elicits cross-reactive antibodies or other immune responses that infer cross-protection, or at least partial cross-protection, against symptomatic and severe dengue. *Plasmodia* have been shown to give rise to polyclonal B-cell activation and to heterophilic antibodies, while some anti-dengue IgM tests have high degree of cross-reactivity with sera from malaria patients. In the following, the historical evolution of falciparum malaria and dengue is briefly reviewed, and we explore early evidence of subclinical dengue in high-transmission malaria areas as well as conflicting reports on severity of co-morbidity. We also discuss examples of other interspecies interactions.

## Present and Past Prevalence of Malaria, Dengue, and Population-Level Protection Against Each Disease

### Origin, Spread, and Burden of Falciparum Malaria

*Plasmodium falciparum* malaria parasites probably emerged as a human pathogen from its enzootic origin in West Africa about 5,000–10,000 years ago (Carter and Mendis, 2002). This coincides with the point in time when humans began living in large agricultural communities, and when African *Anopheles* mosquitoes adapted to the novel agrarian environment breeding in man-made water collections and feeding almost exclusively on humans. The parasite accompanied man to Asia and Melanesia around 4,000 years ago and to the Americas with the slave trade 500 years ago (Curtin, 1993).

According to Carter and Mendis (2002), malaria reached its apex during the 19th century, when more than half of the global population was at risk of infection, and case fatality rates reached above 10%. Declines in malaria incidence were noted by the turn of the 20th century. The halving of the global malaria mortality burden since 2000 has been attributed to a combination of malaria interventions such as the use of insecticide-treated nets (Beiersmann et al., 2011; Kramer et al., 2017), indoor residual spraying, and artemisinin combination therapy (Streatfield et al., 2014), as well as the effect of development, such as increasing urbanization (Tatem et al., 2013) and poverty reduction (Teklehaimanot and Mejia, 2008). Still, each year more than 200 million cases and almost 0.5 million deaths occur due to malaria; these deaths are mainly in children in Sub-Saharan Africa and largely from *Plasmodium falciparum* transmitted by nocturnal *Anopheles* species (Streatfield et al., 2014). Transmission in urban areas is generally of lower intensity than in rural areas but can be surprisingly elevated in some urban environments (Wilson et al., 2015).

No effective vaccine exists for malaria, and natural immunity takes years of repeated exposure to develop. Residents of malaria-endemic areas eventually develop some immunity to the infection (Tomson, 1933), but immunity wanes after a single year without exposure (Hoffman, 1986). The difficulties in developing long lasting immunity, including the mechanism by which partial protection is finally achieved, remain largely unexplained. Two main hypotheses, not mutually exclusive, have been put forward (Greenwood and Targett, 2011). The first suggests that adaptive immunity is especially challenged in the case of *P. falciparum* malaria, as the key parasite protein family, PfEMP1, has more than 60 variants (Gardner et al., 2002). The second postulates that repeated exposure to malaria antigens is needed to drive an effective immune response, perhaps accompanied by a maturation of the immune system with increasing age. Studying inflammatory cytokine responses in individuals with malaria from areas of different transmission intensities have shown that levels of pro-inflammatory cytokines decreased with increasing transmission intensity (Ademolue et al., 2017).

### Origin, Spread, and Burden of Dengue

There is no consensus regarding the enzootic origin of dengue virus (DENV); some suggest an African origin, others an Asian, given the presence of sylvatic (“jungle”) viruses in both regions (Rudnick et al., 1967; Gubler, 1987; Halstead, 1990). Irrespective, the virus probably evolved into four distinct serotypes about 1,000 years ago within sylvatic cycles involving mosquitoes and primates. Phylogenetic evidence suggests that each of the four serotypes entered a cycle of stable transmission between humans and mosquito vectors only 125–320 years ago, coinciding with the out-of-Africa migration of the anthropophilic vector *Aedes aegypti* (Crawford et al., 2017) and historical accounts of local outbreaks and general pandemics of dengue-like disease from the 17th seventeenth century and onwards (Gubler, 1998; Twiddy et al., 2003).

The global pattern of spread of the four dengue serotypes since the early 1940s – when DENV was first isolated – has been highlighted by Messina et al. (2014). These show consistent dispersal of each serotype from South Asia over Southeast Asia to the Pacific and the Americas, with relatively few reported occurrences in sub-Saharan Africa, tribal areas of Papua New-Guinea, North-East India and inner parts of South America. This recently intensified transmission and spread of the DENVs is largely attributed to massive urbanization and globalized travel and trade that characterize the latter half of the 20th century. Importantly, the urban ecology in the tropics has enabled the proliferation of the “domestic” vector *Ae. aegypti*, while travel and trade have helped widen the global range of both vector and virus (Gubler, 1998; Li et al., 2014).

Today, an estimated 3.9 billion people live at risk of infection in at least 128 countries (Brady et al., 2012). Dengue presents a continuously expanding global disease burden, with a suggested doubling or more in the number of symptomatic infections, every 10 years – between 1990 and 2013 (Stanaway et al., 2016). There is, however, wide variation and substantial uncertainty in estimates of the dengue disease burden (Shepard et al., 2014) given both under-recognition and under-reporting of dengue, in general. Notably, only three WHO regions provide regular reports of annual case numbers (WHO, 2017a). In 2015, WHO recoded 3.2 million illness episodes (WHO, 2017a), yet the true figure is probably far higher, with current estimates based on comprehensive risk models ranging between 96 million (Bhatt et al., 2013) in 2010 and 58.4 million in 2013 (Stanaway et al., 2016).

Major challenges for understanding and controlling dengue through the use of preventive or therapeutic interventions include lack of a laboratory correlate and animal models to investigate protective immunity; continued DENV evolution due to major clade replacements and genetic shifts (Holmes and Twiddy, 2003; Rico-Hesse, 2003; Endy, 2014); and not least the complex viral-host pathogenesis that ranges from mild unspecific manifestations to severe and potentially fatal disease (Endy, 2014). A tetravalent vaccine (Capeding et al., 2014; Hadinegoro et al., 2015; Villar et al., 2015) has been licensed for use in several endemic countries in South America and Asia. However, recent data indicate increased risk of severe dengue among vaccinees without previous exposure to DENV (WHO, 2017b) suggesting that – in spite of its tetravalent properties – the vaccine may induce severe manifestations on secondary exposure, through mechanisms of immuno-enhancement (Deen, 2016). Current recommendations by the WHO/GACVS are that individuals who have not been previously infected with wild DENV should not be administered the vaccine (WHO, 2016, 2017b). In addition, the long term effectiveness of this vaccine remains uncertain (López-Gatell et al., 2016). As such, vector control remains the principal dengue prevention option. The primary vectors of DENV, *Ae. aegypti* and *Aedes albopictus*, are both diurnal, rendering insecticide-treated nets of less use for dengue than for malaria control. Public health efforts currently focus on environmental management for elimination of vector breeding sites and on various insecticide applications targeting either larvae or adult stages in the peri-domestic environment (Chu et al., 2013).

### The Emergence of Severe Dengue as an Epidemic Entity

The global emergence of severe DENV infection in the latter half of the 20th century is closely aligned with the intensified transmission and spread of the four serotypes, and the occurrence of sequential transmission or actual co-circulation of two or more serotypes (hyperendemicity) in a given population. Dengue epidemics involving severe disease with mortality rates as high as 10% were first noted in Manila and Bangkok in 1954 (Hammon et al., 1960; Hammon and Sather, 1964), but were soon followed by similar epidemics in several other Southeast Asian cities (Rudnick, 1967; Chambers et al., 1990; Endy, 2014). Urban Southeast Asia remained the focus of intense epidemics of severe dengue throughout the 1960s and 1970s, and by the 1970s severe dengue was recorded as a leading cause of pediatric hospitalization and death in many Southeast Asian countries (WHO, 1997).

Whereas the Pacific islands experienced their first outbreaks with severe dengue in the 1970s, the phenomenon was not reported in the Americas before 1981, when Cuba reported a large-scale epidemic with tens of thousands of severe dengue cases (Guzman and Kouri, 2003). The subsequent pattern of intensified transmission with frequent large-scale epidemics in the urban centers of the Pacific and Americas has largely mimicked that of Southeast Asia, albeit two to three decades later. Thus, in the Americas, dengue has doubled every decade, with increasing severity and mortality particularly in children and young adults (San Martín et al., 2010). Interestingly, Halstead et al. (2001) noted the absence of severe dengue despite hyperendemic DENV transmission in Haiti.

For Sub-Saharan Africa, evidence of dengue disease – not least that of severe disease – has been relatively sparse, even though the disease is likely to have been present for centuries (Amarasinghe et al., 2011). Notably, in 1970, Geser et al. (1970) reported the presence of *Ae. aegypti* in the coastal area of Kenya. In addition, 47% of the population presented with hemagglutinin inhibiting (HI) dengue antibodies, although no outbreaks of dengue had been reported. In 1977, Fagbami et al. found that 45% of tested adults in Nigeria were seropositive for anti-DENV-2 antibodies, as evidenced by neutralizing antibodies (Fagbami et al., 1977). In several African countries DENV transmission has been recognized primarily through diagnosis of travelers or stationed military personnel or through sero-prevalence population studies (de Araújo Lobo et al., 2016; Onoja et al., 2016). It is mainly on the basis of such evidence that dengue is considered endemic to both East and West Africa (Brady et al., 2012).

**FIGURE 1:**
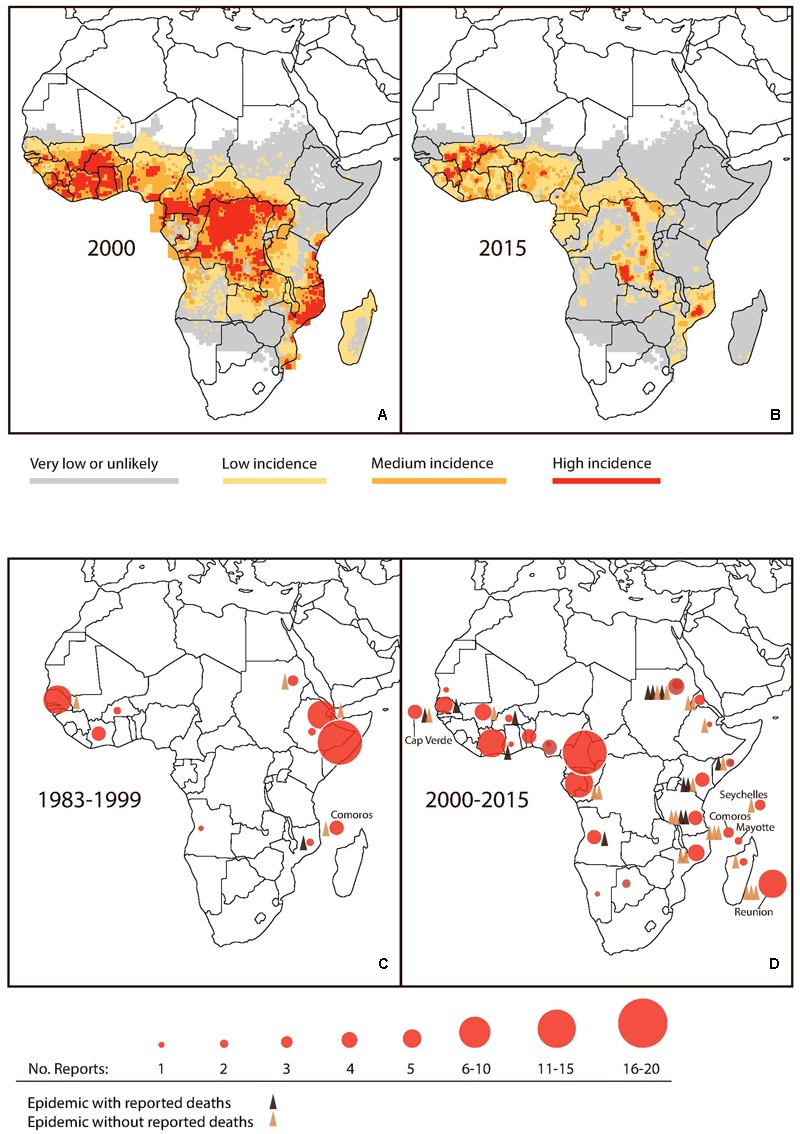
Noted changes in malaria and dengue activity in Sub-Saharan Africa. **(A,B)** Estimated *Plasmodium falciparum* incidence (cases per 100,000) in 2000 and 2015. Modified from original MAP (Source: MAP, 2018). **(C,D)** Accumulated reports of dengue activity and reported dengue epidemics for the periods 1983–1999 and 2000–2015. (Source: Messina et al., 2014; Gideon, 2018).

Recent reviews have cataloged multiple small-scale outbreaks of the 1980s and 1990s, across Sub-Saharan Africa (Messina et al., 2014; Baba et al., 2016). Interestingly, the frequency and scale of outbreaks appear to have increased – as have the numbers of deaths – since the turn of this century (Massangaie et al., 2016). Most notable are: the 2009 outbreak in Cape Verde, with 21,304 reported cases and four deaths; the 2010 outbreak in Sudan involving 3,765 cases and 12 deaths; and the 2011 outbreak in Kenya with 5,000 reported cases and four deaths (Baba et al., 2016). Tanzania has experienced no less than four recurrent outbreaks in 2010, 2012, 2013, and 2014, the latter of which included more than 3,000 reported cases and four deaths (Mboera et al., 2016) (**Figure 1D**).

It is generally argued that dengue and severe dengue have been subject to under-diagnosis in Africa given a lack of index of suspicion and little access to laboratory diagnostics (Amarasinghe et al., 2011). However, awareness is typically high in this region regarding hemorrhagic fevers and shock syndromes due to the recurrent outbreaks of yellow fever, Lassa, Marburg, Ebola and Crimean-Congo hemorrhagic fevers (Parkes-Ratanshi et al., 2014). As such, one would expect that outbreaks involving severe dengue would be subject to substantial levels of scrutiny. In spite of the potential under-diagnosis of dengue, the apparent escalation in dengue epidemic activity in Sub-Saharan Africa (Stanaway et al., 2016) might suggest that the region is on the verge of experiencing the emergence of severe dengue as an epidemic entity, similar to the experience of South and Southeast Asia in the 1950s, the Pacific in the 1970s, and the Americas in the 1980s.

### Risk Factors for Severe Dengue

Several factors have been shown to modulate the clinical response to DENV infection. Notable risk factors for severe manifestations include previous heterotypic DENV infection; viral virulence, co-morbidities such as diabetes and hypertension; as well as host genetic factors (Carabali et al., 2015; Halstead, 2015).

#### Immuno Pathogenesis

The groups of (Halstead, 1980, 1988, 2012), Rothman and Ennis (1999), and Rothman et al. (2014) have both been crucial in detailing different aspects of the immuno-pathological mechanisms of severe disease that arise in response to sequential heterotypic DENV infections. These mechanisms implicate the development of heterotypic, cross-reactive but non-neutralizing antibodies and T cells during primary infection and their subsequent activation upon secondary infections with a heterotypic serotype (Endy, 2014). Cross reactive IgG DENV-antibodies have been shown (*in vitro*) to enhance viral uptake and proliferation in target cells, a mechanism known as antibody dependent enhancement (ADE) of infection with a second dengue serotype. Notably, recent studies suggest that IgG DENV-antibodies may also be linked to thrombocytopenia as observed in severe dengue (Wang et al., 2017). In addition, cross reactive T-cells may unleash a cascade of cytokines and chemical mediators linked to various severity outcomes including endothelial permeability (Pang et al., 2007). New insights in the immunopathology of dengue were recently reviewed by Screaton et al. (2015).

A key point is that the risk of experiencing immune-mediated pathogenesis increases if a population is subject to consecutive infections by heterotypic serotypes. The emergence of severe dengue throughout large parts of the world is thus aligned with the increased global spread of each serotype. Co-circulation of DENV types has been increasingly documented, even in malarious areas of Sub-Saharan Africa (Messina et al., 2014). Although the true extent of DENV co-circulation in Sub-Saharan Africa remains unclear, it is notable that recent outbreaks, as detailed above, have occurred predominantly in areas with reported co-circulation of DENVs (Saluzzo et al., 1986; Caron et al., 2013; Ridde et al., 2016).

#### Virulence

Rapid molecular evolution and genetic diversity is a common feature among RNA viruses and may explain why certain strains and genotypes of the four DENVs display greater epidemic potential and pathogenicity than others. Gubler and Trent (1994) were among the first to argue that increased circulation of DENVs as observed in hyperendemic areas, increases the likelihood of virulent strains, as viral competition intensifies the evolutionary pressure on each serotype.

#### Co-morbidity With Other Than Malaria

A recent review of comorbidity with dengue identified – in addition to old age – several non-communicable diseases of importance for the development of severe manifestations including: cardiovascular disease, diabetes, respiratory disease, and renal disease (Toledo et al., 2016). However, due to heterogeneity among studies, the real estimate effect of comorbidities as modifiers of dengue severity could not be established. Bacterial co-infection with dengue is also increasingly described (Trunfio et al., 2017).

#### Host Genetic Factors

It has been reported that people of African descent are less prone to suffer severe dengue than other ethnic groups, such as Hispanics and Caucasians in Cuba (Guzman and Kouri, 2003), Haiti (Halstead et al., 2001), Brazil (Blanton et al., 2008), and Colombia (Palacios et al., 2016). This observation has offered a plausible explanation for the absence of severe disease from Sub-Saharan Africa – at least up until the recent outbreaks, where severe dengue apparently has increased also among native Africans, as indicated in the following. Furthermore, the study by Moraes et al. (2013) did not confirm that Afro-Americans have lower risks of severe dengue; on the contrary the risk was higher, except when corrected for socio-economic risk factors. HLA and other gene associations have also been linked with dengue disease severity (Stephens, 2010).

## Evidence that Elimination of Falciparum Malaria Coincides with the Emergence of Severe Dengue

In a review of the malaria elimination history, Tanner and de Savigny (2008) noticed how regional malaria elimination campaigns conducted in the late 1940s paved the way for the Global Malaria Eradication Program in 1955. As part of this campaign malaria was eliminated from Europe, North America, the Caribbean (except Haiti) (Agarwal et al., 2012) and parts of Asia and South and Central America during the 1960s and by the early 1970s (Bruce-Chwatt, 1986).

After a temporary setback, in part due to the development of insecticide- and drug-resistance, reduction of malaria by 50–75% was eventually obtained in most of Asia and South and Central America (WHO, 2013). However, no major success was obtained in Sub-Saharan Africa until recent decades. As indicated by **Figures 1A,B**, malaria is now on the retreat in several parts of sub-Saharan Africa. Since 2000, a historically unprecedented decline has been observed (Snow et al., 2017, the Malaria Atlas Project - MAP). For example, malaria has virtually disappeared even in children in Dar es Salaam, Tanzania (Strøm et al., 2013). At the same time, major outbreaks of dengue have been reported in Dar es Salaam, including the 2014 DENV2 outbreak registering a case fatality rate of 4% (Vairo et al., 2016). Dengue epidemics have also been reported from other parts of Tanzania (Mboera et al., 2016), neighboring Kenya (Brady et al., 2012), Mozambique (Massangaie et al., 2016), as well as Angola (Sharp et al., 2015), Sierra Leone (de Araújo Lobo et al., 2016), Burkina Faso (Ridde et al., 2016), and Nigeria (Onoja et al., 2016) and several other parts of Sub-Saharan Africa (L’Azou et al., 2015). Notably, in 2017, dengue outbreaks were reported from eight Sub-Saharan countries reaching an excess of 21,000 total cases. Burkina Faso recorded more than half of these cases (13,135) including 28 deaths (Gideon, 2018). Details for accumulated reports of dengue including epidemic activity in Sub-Saharan Africa from 1983 to 2015 are provided in **Figures 1B,C**.

## Co-Morbidity of Dengue and Malaria

So far, we have discussed concurrent phenomena of dengue emergence and malaria decline at population-level. Another way to consider the hypothesis of mutual exclusion or interdependence of the two pathogens comes from evidence of co-infections at the patient-level. Clinical diagnosis and distinction of malaria and dengue is difficult due to overlapping symptoms. Either disease may induce similar general symptoms as well as severe manifestations in terms of thrombocytopenia, central nervous symptoms, “cytokine-storm” (Clark, 2007), shock and even bleeding. Manifestations of concurrent infection in a patient would be expected to have severe consequences (Hati et al., 2012). However, as noticed by Mohapatra et al. (2012) reports on co-infection are relatively scarce in literature and those of severe manifestations even more so.

The first patient with falciparum malaria and dengue co-infection was reported by Charrel et al. (2005). Our own search from 2005 to 2017 revealed 55 reports of co-infections, including more than 360 cases of acute co-infections as confirmed by malaria RDT and/or microcopy and DENV virology, antigen tests and/or seroconversion (for details please refer to Supplementary Material). The malaria-immune status was not reported for most co-infections neither were parasitaemia levels which could otherwise be used as a proxy for immunity (with high parasitaemia indicating low immunity). Instead, we suggest that malaria incidence in the countries from where co-infections were reported may be used to assume the general level of malaria immunity. With the exception of Sub-Saharan Africa, most areas reported low or medium transmission of *P. falciparum* malaria during the period of interest.

**Table 1** includes only cases of *P. falciparum* in acute dengue confirmed by DENV RT-PCR, NS1 test, or seroconversion. Of 74 identified co-infections, severe symptoms were only recorded in three cases of which one was acquired in West Africa as reported by Charrel et al. (2005). This case involved a traveler, assumed to be non-malaria immune, who after returning to France developed disseminated intravascular coagulation, which may be a manifestation of either disease (*ibid*). Alam and Dm (2013) reported a single case of cerebral malaria and dengue co-infection in a military personnel located in Patna, India. Again, the level of parasitaemia was not reported; however, cerebral malaria would indicate low or absence of malaria immunity, in line with the low incidence level reported for the area. Finally, Yong et al. (2012) reported severe manifestations in a local Indonesian adult male with 4% falciparum parasitaemia and dengue co-infection. This case was complicated by rhabdomyolysis and acute renal insufficiency, which may both be induced by malaria and dengue.

**Table 1:** Reported cases of acute dengue virus and *P. falciparum* co-infections by global region, 2005–2017.

Region	Total cases	Mild symptoms	Severe symptoms	Severity not reported
Sub-Saharan Africa	5	1	1^1^	3
Americas	28	7	0	21
South Asia	39	38	1^2^	0
Southeast Asia	2	1	1^3^	0
Total	74	47	3	24

*Method of confirmation of acute dengue: RT-PCR, NS1 test and/or IgM/IgG seroconversion. Categorization of severity based on case description by source authors. Sources for severe cases: ^*1*^Charrel et al., 2005 (returning traveller); ^*2*^Alam and Dm, 2013 (military personnel; ^*3*^Yong et al., 2012 (local resident). For additional sources please refer to Supplementary Table 1).*

Lack of reporting of dual infection in the field could result from testing positive for either malaria or dengue infection with no further testing being done, given low index of suspicion for co-infections (i.e., case considered diagnosed after one positive test). Yet, some argue that severity could also prompt testing for dual disease, and thereby potentially bias reporting. Notably, studies that have actively investigated the clinical picture of mono-infections as compared to that of dengue and malaria co-infections disagree with respect to observed differences in severity levels. Epelboin et al. (2012) reported that among local resident in French Guyana, the clinical picture of dual infections was more severe than in either dengue or malaria alone. It is notable that *P. vivax* was identified in the majority of cases (∼80%) and that only 50% of the dual infected reported previous malaria. Unfortunately, the report did not stratify between *P. vivax* or *P. falciparum* co-infections, nor did it report on parasitaemia levels.

In contrast, Halsey et al. (2016) found similar severity levels between mono and co-infected individuals among local populations in Peru. As for Epelboin et al. (2012) the majority of cases were reported as *P. vivax*. In India, Mohapatra et al. (2012) reported generally benign manifestations among co-infections as compared with malaria mono-infections. In this study the majority of cases (∼90%) were reported as *P. falciparum* infections. Notably, lower parasitic counts were observed among co-infected than for mono-infected patient, although reported as non-significant. In line with these findings, Ahmad reported that the presentation and severity of co-infections appeared “to be milder and outcomes comparable (if not better) to mono-infections” during a dengue outbreak in Uttarakhand, India. While co-infections occurred with both *P. falciparum* and *P. vivax* these were not clearly distinguished in the report.

Stoler et al. (2015) reported dengue-specific IgG antibodies in 22% of urban Ghanaian children with *P. falciparum* parasitaemia, suggesting previous exposure, and dengue IgM antibodies in 3%, indicating probable acute co-infection. While co-infection was indicated, co-morbidity or increased severity was not. None of the children appeared to have severe disease; but IgM dengue positive children had (non-significantly) higher levels of *P. falciparum* parasitaemia, which could indicate they were only partially immune to falciparum malaria at the time of DENV infection. Unfortunately, levels of malaria antibodies were not reported. Specificity of the anti-dengue Ig tests used was 97% for multisite pooled sera, though cross-reactivity with other flavivira was high (30% and 60% for IgM and IgG, respectively).

### Cross-Reactivity and “False-Positivity” in Tests for Dengue and Malaria

Malaria infection gives rise to polyclonal B-cell activation (Greenwood, 1974) and to heterophilic antibodies (Houba et al., 1974), which may react to host antigens as well as to other pathogens (Ribeiro, 1988). Some anti-dengue IgM tests have shown a high degree of cross-reactivity (10–70%) with sera from malaria patients, and with other flavivira (Hunsperger et al., 2009; WHO, 2009). Hunsperger et al. (2009), when evaluating four anti-DENV IgM kits and five micro-plate ELISAs found between 5 and 70% false-positive results for patients with malaria or past dengue infections. Knowing the true population prevalence of dengue as well as malaria infections is hampered by the lack of representative population-based studies, and also by false-positive test results or cross-reactivity that are a problem in many serological assays including rapid diagnostic tests (RDTs) for dengue and malaria. In other words, a positive DENV IgM test may signify current or recent primary infection, secondary or tertiary infection (often lower titers, though primary infection responses also vary), or cross-reactivity with other virus or even malaria (current or past infection). Thus, with current laboratory assays available in countries with limited resources, it is very difficult to tease apart the possibility of malaria and dengue together in an area or a patient, wherefore low-cost antigen tests and viral and plasmodia gene detection kits are needed.

### Possible Co-protection of Malaria Infection Against Severe Dengue?

Repeated exposure to falciparum malaria is followed by development of protective antibodies, decline of pro-inflammatory host-responses (Ademolue et al., 2017) and decreasing severity (Reyburn et al., 2005), whereas sequential exposure to heterogeneous DENVs – and related viruses, e.g., Zika virus – may lead to enhanced host immune responses (Cohen, 2017). However, on the basis of the presented clinical, epidemiological, immunological and laboratory-based reports, we speculate whether cross-reactivity observed between tests for dengue and malaria pathogens could also be an indicator of some level of cross-protection between two potentially severe diseases. In other words, could dengue-induced antibody-dependent enhancement of infection (ADE) (de Alwis et al., 2014) be “de-hanced” by malaria induced antibodies? Or could alleviated cytokine responses among clinically malaria immune individuals (Ademolue et al., 2017) similarly reduce “over”-reaction to dengue (re-)infection?

Proof of concept of cross-protection from malaria to dengue in man would require exposing malaria immune humans to dengue in controlled experiments. As reviewed by Sabin (1952), research on dengue during World War II did include research on human volunteers exposed to DENV and the development of immunity to the homologues strain. Unfortunately, the level of immunity to malaria was not reported in these volunteers. A human model of dengue exposure is currently being re-introduced (Larsen et al., 2015; Royer, 2015).

### Examples of Interspecies Interactions

In the literature, there are certainly important examples of interaction between infections of unrelated pathogens leading to more severe outcomes, such as HIV infection leading to latent tuberculosis reactivation (Pitchenik et al., 1983), and severe outcomes of pneumococcal disease following influenza infection, the so-called lethal synergy (McCullers, 2014). And most recently, Zika virus infection has been found to be enhanced in recent dengue cases, much like ADE among the four DENV types (Kawiecki and Christofferson, 2016). But examples in which co- or sequential infection may decrease severity of either or both diseases have been less investigated. An interesting, historical example is the now obsolete treatment of syphilis with malaria (Moore, 1930), and the possible sharp reduction in tuberculosis in the US population immediately following the 1918 severe pandemic influenza outbreak (Noymer, 2011). More recently, it has been shown that *P. falciparum* infection may protect partly against severe Ebola outcomes (Rosenke et al., 2016).

## Conclusion

In summary, we conclude that at the population level the evidence of interaction between dengue and malaria is perhaps already there: when comparing time trends in the two diseases at continent level, a suggestive pattern emerges. We have discussed the striking coincidence of the emergence of dengue in recent years (**Figure 1D**), with the reduction in incidence of malaria (**Figure 1B**), suggesting that declining malaria exposure due to successful control programs or other environmental factors may have inadvertently led to the cessation of protection against clinically apparent and severe dengue infections, especially in Sub-Saharan Africa. However, individual-level studies would be the best way to determine if co-protection exists between falciparum malaria and dengue. Available individual-level studies reviewed here are only suggestive and interpretation hampered by non-specificity of used tests. Also, they present a risk of bias in favor of our hypothesis, in that more severe patients are more likely to be tested for several pathogens and subsequently reported, and vice versa that lack of reporting of dual infection in the field could result from testing positive for either malaria or dengue infection and then no further testing being done.

Evidently, more research is required to investigate whether there is a causal link between declining falciparum malaria and increased dengue in recent years. We suggest *in vitro* experiments such as exposing *in vitro* cultures of DENV to malaria antibodies, and vice versa as a proxy to evidence (or not) of cross-protection. Other modern *in vitro* technologies including epigenetic silencing or activation of regulatory genes implicated in immunity and susceptibility of disease (Kirchner et al., 2016) or development of animal models may also help, while acknowledging that suitable animal models are still not available for either infection. It would be interesting – if considered ethically acceptable – to include malaria-immune individuals in the ongoing dengue human exposure model. Further studies of the potential role of unspecific sides of acquired immunity (Muraille, 2016), and studies of cross-kingdom interaction (Luckhart et al., 2015) could be rewarding as well. Especially, if combined with integrated epidemiology and surveillance for vector-borne diseases and by integrating layers of data from both malaria and dengue (Wardrop, 2016). These studies could be complemented by large-scale prospective studies e.g. through the INDEPTH-network of health and demographic surveillance systems in low- and middle-income countries (INDEPTH, 2018).

Finally, we believe it is important to broaden considerations across pathogens and diseases in order to explain the striking associations in temporal patterns of malaria and dengue globally observed since the 1950s and in Africa, specifically, after the Millennium. More broadly, we suggest that considering non-specific effects of inter-species interaction and interdependences may be a critical aspect of recognizing possible outcomes beyond those of the target pathogen and to understand the broader consequences of global health intervention programs designed to eliminate a given disease.

## Author Contributions

IB conceived the hypothesis of potential interaction between malaria and dengue, and drafted the first version of the manuscript, and wrote the subsequent versions in close collaboration with KS and LS. KS contributed in particular to the sections of dengue and malaria epidemiology mapping, and to **Table 1** and to the Supplementary Material, and contributed to all sections and versions of the manuscript. LS contributed in particular to the sections on historic epidemiology and interspecies interaction and to other sections and versions of the manuscript. All authors read and approved the submitted version.

## Conflict of Interest Statement

The authors declare that the research was conducted in the absence of any commercial or financial relationships that could be construed as a potential conflict of interest.
