# Supplementary material for: Elimination of Falciparum Malaria and Emergence of Severe Dengue: An Independent or Interdependent Phenomenon?
Source: Front Microbiol. 2018 May 30;9:1120. doi: 10.3389/fmicb.2018.01120 (PMC5989664; doi:10.3389/fmicb.2018.01120)
Supplement: Supplementary file 1 [file Table_1.docx]

Supplementary Table 1. Reported cases of dengue and malaria co-infections identified for the period 1995-2017.

| Country of infection | Study Design | Year of reported infection | *P. falciparum* & DENV | | | *P. vivax* & DENV | | | *P. falciparum* & *P. vivax* & DENV | Other Plasmodium species or unreported + DENV | Identified blood specimen | DENV infection status | Notes | Study ID |
| --- | --- | --- | --- | --- | --- | --- | --- | --- | --- | --- | --- | --- | --- | --- |
|  |  |  | All cases | Non severe | Severe | All cases | Non severe | Severe |  |  |  |  |  |  |
| Bangladesh | Surveillance study | 2008-9 | - | - | - | 1 | 0 | 1 | - | - | IgM | Probable acute | Four-year-old local boy | (Faruque et al., 2012) |
| Bangladesh | Cross-sectional study | - | - | - | - | - | - | - | - | 19 | IgM | Probable acute |  | (Swoboda et al., 2014) |
| Brazil | Cross-sectional study | - | - | - | - | 2 | 2 | 0 | - | - | DENV-2 | Acute |  | (Santana et al., 2010) |
| Brazil | Cross-sectional study | 2009-11 | - | - | - | 44 | 12 | 32 | - | - | DENV; NS1; IgM | Acute | Severity not specified among 27 acute cases | (Magalhaes et al., 2014) |
| Brazil | Case report | 2009-13 | - | - | - | 30 | - | - | - | - | DENV1-4; NS1; IgM | Acute |  | (Mendonca et al., 2015) |
| Brazil | Case report | - | - | - | - | - | - | - | 1 | 1 | NS1; IgM; IgG | Acute | *P. ovale* | (Lupi et al., 2016) |
| Cambodia | Surveillance study | 2006-9 | - | - | - | - | - | - | - | 15 | DENV | Acute | *Pf* or *Pv* co-infection with DENV | (Kasper et al., 2012) |
| Cambodia | Surveillance study | 2008-10 | - | - | - | - | - | - | - | 27 | DENV | Acute | Non-severe *Pf* or *Pv* co-infection with DENV | (Mueller et al., 2014) |
| French Guyana | Retrospective study | 2004-5 | 3 | 3 | 0 | 14 | 14 | 0 | - | - | DENV-3; IgM | Acute | 1 of 14 cases probable acute | (Carme et al., 2009) |
| French Guyana | Case Series | 2009-10 |  |  |  | 11 | 1 | 10 | - | - | DENV-2,3,4 | Acute | 1 case DENV3+4 co-infection | (Magalhaes et al., 2012) |
| French Guyana | Case control (Matched-pair) | 2004-10 | 21 | - | - | 80 | - | - | 3 | - | DENV1-4; IgA; IgM; NS1 | Acute |  | (Epelboin et al., 2012) |
| Ghana | Cross-sectional study | 2011-14 | 7 | 7 | 0 | - | - | - | - | - | IgM; IgG | Probable acute |  | (Stoler et al., 2015) |
| Guinea/Senegal/Sierra Leone | Case report | 2004 | 1 | 0 | 1 | - | - | - | - | - | IgM; IgG; DENV-3 | Acute | Returning traveller | (Charrel, Brouqui, Foucault, & de Lamballerie, 2005) |
| Haiti | Case report | 2011 | 1 | 1 | 0 | - | - | - | - | - | DENV-4; IgG | Acute | Returning traveller | (Serre et al., 2015) |
| India | Case report | 2003 | 2 | 2 | 0 | - | - | - | - | - | IgM; IgG | Probable acute |  | (C Arya et al., 2005) |
| India | Surveillance study | 2003-4 | 1 | 0 | 1 | 8 | - | - | - | - | IgM | Probable acute | 1 fatal case | (Ali, Nadeem, Anwar, Tariq, & Chotani, 2006) |
| India | Case report | 2003 | - | - | - | 1 | 1 | 0 | - | - | IgM; IgG | Probable acute | Returning traveller | (Deresinski, 2006) |
| India | Case report | - | 1 | 1 | 0 | - | - | - | - | - | IgM | Probable acute |  | (Bhalla, Sharma, Sharma, & Suri, 2006) |
| India | Case report | - | - | - | - | 1 | 1 | 0 | - | - | DENV-2 | Acute |  | (Thangaratham, Jeevan, Rajendran, Samuel, & Tyagi, 2006) |
| India | Case report | - | 1 | 0 | 1 | - | - | - | - | - | IgM | Probable acute | 1 fatal case | (Ward, 2006) |
| India | Case report | - | 1 | 1 | 0 | - | - | - | - | - | IgM; IgG | Acute | *Pf* and *Pv* co-infection with DENV. Acute dengue confirmed by seroconversion | (Kaushik, Varma, Kaushik, & Gaur, 2007) |
| India | Case report | - | 1 | 0 | 1 | - | - | - | - | - | IgM | Probable acute |  | (Chander, Singla, & Singh, 2009) |
| India | Surveillance study | 2007-9 | 6 | 0 | 6 | - | - | - | - | - | IgM | Probable acute |  | (Bhaskar, Moorthy, Kumar, & Arthur, 2010) |
| India | Surveillance study | 2011 | 24 | 23 | 1 | 2 | 2 | 0 | 1 | - | NS1; IgM | Acute | Low parasitic count observed for co-infections as opposed to malaria mono-infections | (Mohapatra, Patra, & Agrawala, 2012) |
| India | Case report | 2012 | - | - | - | 1 | 1 | 0 | - | - | NS1; IgM | Acute |  | (Malhotra, 2012) |
| India | Surveillance study | 2005-10 | 18 | - | - | 28 | - | - | - | - | IgM; IgG | Probable acute |  | (Hati et al., 2012) |
| India | Case report | 2012 | - | - | - | 1 | 1 | 0 | - | - | IgM | Probable acute | Returning traveller | (Mushtaq, Qadri, & Rashid, 2013) |
| India | Case report | - | 1 | 0 | 1 | - | - | - | - | - | IgM; IgG | Acute |  | (Alam & Dm, 2013) |
| India | Case report | - | - | - | - | 1 | 1 | 0 | - | - | NS1; IgM; IgG | Acute |  | (Pande & Guharoy, 2013) |
| India | Case report | - | - | - | - | 1 | 1 | 0 | - | - | DENV1,4 | Acute | Sickle cell co-morbidity | (Barde, Jatav, Bharti, Godbolea, & Singha, 2013) |
| India | Case report | - | 1 | 1 | 0 | - | - | - | - | - | IgM | Probable acute |  | (Suresh, Krishna, Raju, Teja, & Usha, 2013) |
| India | Case series | - | - | - | - | 3 | 3 | 0 | - | - | IgM; IgG | Acute | Acute dengue cases confirmed by seroconversion | (Bhagat, Kanhere, Phadke, & George, 2014) |
| India | Case report | - | - | - | - | 1 | 0 | 1 | - | - | NS1; IgM | Acute | *Pf* , DENV-1 and scrub typhus co-infection | (Kumar et al., 2014) |
| India | Case report | - | 1 | 1 | 0 | - | - | - | - | - | NS1; IgM | Acute |  | (Satyawali, Pandey, Rawat, & Khalil, 2014) |
| India | Case report | - | - | - | - | 1 | 1 | 0 | - | - | NS1; IgM | Acute | *Pv*, DENV and Hepatitis A. co-infection. Recent travel history | (Tulara, 2015) |
| India | Surveillance study | 2013 | 12 | 12 | 0 | 10 | 10 | 0 | - | - | DENV; NS1; IgM; IgG | Acute |  | (Rao, Padhy, & Das, 2016) |
| India | Case report | - | - | - | - | - | - | - | 1 | - | IgM | Probable acute |  | (Saksena et al., 2017) |
| India | Retrospective study | 2014-15 | - | - | - | - | - | - | - | 44 | NS1; IgM | Acute |  | (Barua & Yeolekar, 2016) |
| India | Cross-sectional study | 2013-14 | - | - | - | - | - | - | - | 3 | Not specified | Uncertain |  | (Raja, Mary, & Usha, 2016) |
| India | Retrospective study | 2012-13 | - | - | - | - | - | - | 1 | 9 | NS1; IgM; IgG | Acute |  | (Ahmad et al., 2016) |
| Indonesia | Case report | - | 1 | 0 | 1 | - | - | - | - | - | IgG | Previous dengue |  | (Thaha, Pranawa, Yogiantoro, Tanimoto, & Tomino, 2008) |
| Indonesia | Case report | - | 1 | 0 | 1 | - | - | - | - |  | IgM | Acute | Acute dengue confirmed by seroconversion | (K. P. Yong, Tan, & Low, 2012) |
| Jamaica | Surveillance study | 2007-8 | - | - | - | - | - | - | - | 74 | IgM | Probable acute | 4 cases of *P,* DENV and leptospirosis co-infection | (Lindo et al., 2013) |
| Malaysia | Case report | - | - | - | - | 1 | 1 | 0 | - | - | IgM | Probable acute |  | (L. S. Yong & Koh, 2013) |
| Malaysia–Thailand | Case report | - | - | - | - | - | - | - | 1 | 1 | NS1; IgM | Acute | *Pf, Pv* and DENV co-infection. Severe case | (Chong, Mohamad Zaini, Suraiya, Lee, & Lim, 2017) |
| Nigeria | Cohort study | 2008 | 18 | - | - | - | - | - | - | - | PRNT | Probable acute |  | (Baba et al., 2013) |
| Nigeria | Case report |  | 1 | 1 | 0 | - | - | - | - | - | DENV | Acute | Pf, DENV and CHIKV Co-infection | (Raut, Rao, Sinha, Hanumaiah, & Manjunatha, 2015) |
| Nigeria | Surveillance study | 2014 | 1 | 1 | 0 | - | - | - | - | - | NS1; IgM; IgG | Probable acute |  | (Ayorinde, Oyeyiga, Nosegbe, & Folarin, 2016) |
| Nigeria | Surveillance study | - | 11 | 11 | 0 | - | - | - | - | 4 | DENV-2,3,4; IgM; IgG | Acute |  | (Kolawole, Seriki, Irekeola, Bello, & Adeyemi, 2017) |
| Pakistan | Case series | 2007-8 | 1 | - | - | 25 | - | - | - | - | IgM; IgG | Probable acute |  | (Abbasi et al., 2009) |
| Pakistan | Cross-sectional study | 2012 | 3 | 3 | 0 | 15 | 14 | 1 | - | - | IgM | Probable acute |  | (Assir, Masood, & Ahmad, 2014) |
| Pakistan | Cross-sectional study | - | - | - | - | - | - | - | - | 5 | IgM | Probable acute |  | (Yasir, Rashid, Moin, & Owais, 2014) |
| Peru | Surveillance study + Case-match | 2002-11 | 3 | 3 | 0 | 14 | 14 | 0 | - | - | DENV-1, 3 | Acute |  | (Halsey et al., 2016) |
| Senegal | Surveillance study | 2009-13 | 3 | - | - | - | - | - | - | - | DENV | Acute |  | (Sow et al., 2016) |
| Thailand | Case report | - | 1 | 1 | 0 | - | - | - | - | - | NS1; IgM | Acute |  | (Issaranggoon na ayuthaya, Wangjirapan, & Oberdorfer, 2014) |
| **SUB TOTAL** | | | 147 | 72 | 13 | 296 | 80 | 45 | 11 | 198 |  |  |  |  |
| **TOTAL (all dengue and malaria co-infections)** | | | 652 |  |  |  |  |  |  |  |  |  |  |  |

References

Abbasi, A., Butt, N., Sheikh, Q. H., Bhutto, A. R., Munir, S. M., & Ahmed, S. M. (2009). Clinical Features, Diagnostic Techniques and Management of Dual Dengue and Malaria Infection. *Jcpsp-Journal of the College of Physicians and Surgeons Pakistan, 19*(1), 25-29.

Ahmad, S., Dhar, M., Mittal, G., Bhat, N., Shirazi, N., Kalra, V., . . . Gupta, V. (2016). A comparative hospital-based observational study of mono-and co-infections of malaria, dengue virus and scrub typhus causing acute undifferentiated fever. *European Journal of Clinical Microbiology & Infectious Diseases, 35*(4), 705-711.

Alam, A., & Dm, M. (2013). A case of cerebral malaria and dengue concurrent infection. *Asian Pac J Trop Biomed, 3*(5), 416-417. doi:10.1016/S2221-1691(13)60087-8

Ali, N., Nadeem, A., Anwar, M., Tariq, W. U., & Chotani, R. A. (2006). Dengue fever in malaria endemic areas. *J Coll Physicians Surg Pak, 16*(5), 340-342. doi:5.2006/JCPSP.340342

Assir, M. Z. K., Masood, M. A., & Ahmad, H. I. (2014). Concurrent dengue and malaria infection in Lahore, Pakistan during the 2012 dengue outbreak. *International Journal of Infectious Diseases, 18*, 41-46.

Ayorinde, A. F., Oyeyiga, A. M., Nosegbe, N. O., & Folarin, O. A. (2016). A survey of malaria and some arboviral infections among suspected febrile patients visiting a health centre in Simawa, Ogun State, Nigeria. *J Infect Public Health, 9*(1), 52-59. doi:10.1016/j.jiph.2015.06.009

Baba, M., Logue, C. H., Oderinde, B., Abdulmaleek, H., Williams, J., Lewis, J., . . . P, D. A. (2013). Evidence of arbovirus co-infection in suspected febrile malaria and typhoid patients in Nigeria. *J Infect Dev Ctries, 7*(1), 51-59. doi:10.3855/jidc.2411

Barde, P., Jatav, J., Bharti, P., Godbolea, S., & Singha, N. (2013). Concomitant infection of dengue virus serotypes and malaria in a sickle cell disease patient: a case-study. *Dengue, 37*, 223.

Barua, A., & Yeolekar, M. E. (2016). Concurrent dengue and malaria coinfection: Observations from a central Mumbai hospital. *International Journal of Infectious Diseases, 45*, 165. doi:<https://doi.org/10.1016/j.ijid.2016.02.393>

Bhagat, M., Kanhere, S., Phadke, V., & George, R. (2014). Concurrent malaria and dengue fever: a need for rapid diagnostic methods. *Journal of family medicine and primary care, 3*(4), 446.

Bhalla, A., Sharma, N., Sharma, A., & Suri, V. (2006). Concurrent infection with dengue and malaria. *Indian J Med Sci, 60*(8), 330-331.

Bhaskar, M. E., Moorthy, S., Kumar, N. S., & Arthur, P. (2010). Dengue haemorrhagic fever among adults – An observational study in Chennai, south India. *The Indian Journal of Medical Research, 132*(6), 738-740.

C Arya, S., K Mehta, L., Agarwal, N., Agarwal, B. K., Mathai, G., & Moondhara, A. (2005). Episodes of Concurrent Dengue and Malaria.

Carme, B., Matheus, S., Donutil, G., Raulin, O., Nacher, M., & Morvan, J. (2009). Concurrent dengue and malaria in Cayenne Hospital, French Guiana. *Emerg Infect Dis, 15*(4), 668-671. doi:10.3201/eid1504.080891

Chander, J., Singla, N., & Singh, R. (2009). Concurrent presence of dengue and Plasmodium falciparum. *Tropical medicine and health, 37*(2), 69-70.

Charrel, R. N., Brouqui, P., Foucault, C., & de Lamballerie, X. (2005). Concurrent dengue and malaria. *Emerg Infect Dis, 11*(7), 1153-1154. doi:10.3201/eid1107.041352

Chong, S. E., Mohamad Zaini, R. H., Suraiya, S., Lee, K. T., & Lim, J. A. (2017). The dangers of accepting a single diagnosis: case report of concurrent Plasmodium knowlesi malaria and dengue infection. *Malar J, 16*(1), 2. doi:10.1186/s12936-016-1666-y

Deresinski, S. (2006). Concurrent plasmodium vivax malaria and dengue. *Emerg Infect Dis, 12*(11), 1802. doi:10.3201/eid1211.060341

Epelboin, L., Hanf, M., Dussart, P., Ouar-Epelboin, S., Djossou, F., Nacher, M., & Carme, B. (2012). Is dengue and malaria co-infection more severe than single infections? A retrospective matched-pair study in French Guiana. *Malar J, 11*, 142. doi:10.1186/1475-2875-11-142

Faruque, L. I., Zaman, R. U., Alamgir, A. S., Gurley, E. S., Haque, R., Rahman, M., & Luby, S. P. (2012). Hospital-based prevalence of malaria and dengue in febrile patients in Bangladesh. *Am J Trop Med Hyg, 86*(1), 58-64. doi:10.4269/ajtmh.2012.11-0190

Halsey, E. S., Baldeviano, G. C., Edgel, K. A., Vilcarromero, S., Sihuincha, M., & Lescano, A. G. (2016). Symptoms and Immune Markers in Plasmodium/Dengue Virus Co-infection Compared with Mono-infection with Either in Peru. *PLoS Negl Trop Dis, 10*(4), e0004646. doi:10.1371/journal.pntd.0004646

Hati, A. K., Bhattacharjee, I., Mukherjee, H., Bandyopadhayay, B., Bandyopadhyay, D., De, R., & Chandra, G. (2012). Concurrent dengue and malaria in an area in Kolkata. *Asian Pac J Trop Med, 5*(4), 315-317. doi:10.1016/S1995-7645(12)60046-7

Issaranggoon na ayuthaya, S., Wangjirapan, A., & Oberdorfer, P. (2014). An 11-year-old boy with Plasmodium falciparum malaria and dengue co-infection. *BMJ Case Rep, 2014*. doi:10.1136/bcr-2013-202998

Kasper, M. R., Blair, P. J., Touch, S., Sokhal, B., Yasuda, C. Y., Williams, M., . . . Putnam, S. D. (2012). Infectious etiologies of acute febrile illness among patients seeking health care in south-central Cambodia. *Am J Trop Med Hyg, 86*(2), 246-253. doi:10.4269/ajtmh.2012.11-0409

Kaushik, R. M., Varma, A., Kaushik, R., & Gaur, K. J. (2007). Concurrent dengue and malaria due to Plasmodium falciparum and P. vivax. *Trans R Soc Trop Med Hyg, 101*(10), 1048-1050. doi:10.1016/j.trstmh.2007.04.017

Kolawole, O. M., Seriki, A. A., Irekeola, A. A., Bello, K. E., & Adeyemi, O. O. (2017). Dengue virus and malaria concurrent infection among febrile subjects within Ilorin metropolis, Nigeria. *J Med Virol, 89*(8), 1347-1353. doi:10.1002/jmv.24788

Kumar, S., Kumar, P. S., Kaur, G., Bhalla, A., Sharma, N., & Varma, S. (2014). Rare concurrent infection with scrub typhus, dengue and malaria in a young female. *J Vector Borne Dis, 51*(1), 71-72.

Lindo, J., Brown, P. D., Vickers, I., Brown, M., Jackson, S. T., & Lewis-Fuller, E. (2013). Leptospirosis and malaria as causes of febrile illness during a dengue epidemic in Jamaica. *Pathog Glob Health, 107*(6), 329-334. doi:10.1179/2047773213Y.0000000112

Lupi, O., Ridolfi, F., da Silva, S., Zanini, G. M., Lavigne, A., Nogueira, R. M., . . . Brasil, P. (2016). Dengue infection as a potential trigger of an imported Plasmodium ovale malaria relapse or a long incubation period in a non-endemic malaria region. *Int J Infect Dis, 44*, 20-24. doi:10.1016/j.ijid.2016.01.008

Magalhaes, B. M., Alexandre, M. A., Siqueira, A. M., Melo, G. C., Gimaque, J. B., Bastos, M. S., . . . Mourao, M. P. (2012). Clinical profile of concurrent dengue fever and Plasmodium vivax malaria in the Brazilian Amazon: case series of 11 hospitalized patients. *Am J Trop Med Hyg, 87*(6), 1119-1124. doi:10.4269/ajtmh.2012.12-0210

Magalhaes, B. M., Siqueira, A. M., Alexandre, M. A., Souza, M. S., Gimaque, J. B., Bastos, M. S., . . . Mourao, M. P. (2014). P. vivax malaria and dengue fever co-infection: a cross-sectional study in the Brazilian Amazon. *PLoS Negl Trop Dis, 8*(10), e3239. doi:10.1371/journal.pntd.0003239

Malhotra, V. (2012). Concurrent malaria and dengue infection. *International Journal of Health & Allied Sciences, 1*(3), 181-182. doi:10.4103/2278-344x.105083

Mendonca, V. R., Andrade, B. B., Souza, L. C., Magalhaes, B. M., Mourao, M. P., Lacerda, M. V., & Barral-Netto, M. (2015). Unravelling the patterns of host immune responses in Plasmodium vivax malaria and dengue co-infection. *Malar J, 14*, 315. doi:10.1186/s12936-015-0835-8

Mohapatra, M. K., Patra, P., & Agrawala, R. (2012). Manifestation and outcome of concurrent malaria and dengue infection. *J Vector Borne Dis, 49*(4), 262-265.

Mueller, T. C., Siv, S., Khim, N., Kim, S., Fleischmann, E., Ariey, F., . . . Menard, D. (2014). Acute undifferentiated febrile illness in rural Cambodia: a 3-year prospective observational study. *PLoS One, 9*(4), e95868. doi:10.1371/journal.pone.0095868

Mushtaq, M. B., Qadri, M. I., & Rashid, A. (2013). Concurrent infection with dengue and malaria: an unusual presentation. *Case Rep Med, 2013*, 520181. doi:10.1155/2013/520181

Pande, A., & Guharoy, D. (2013). A case report of Plasmodium vivax, Plasmodium falciparum and dengue co-infection in a 6 months pregnancy. *Ann Med Health Sci Res, 3*(Suppl 1), S16-17. doi:10.4103/2141-9248.121211

Raja, J. M., Mary, A., & Usha, S. (2016). A Study on Dual Infections in Pyrexia Cases. *Health Sciences, 5*(8), 150-155.

Rao, M. R., Padhy, R. N., & Das, M. K. (2016). Prevalence of dengue viral and malaria parasitic co-infections in an epidemic district, Angul of Odisha, India: An eco-epidemiological and cross-sectional study for the prospective aspects of public health. *J Infect Public Health, 9*(4), 421-428. doi:10.1016/j.jiph.2015.10.019

Raut, C. G., Rao, N. M., Sinha, D. P., Hanumaiah, H., & Manjunatha, M. J. (2015). Chikungunya, dengue, and malaria co-infection after travel to Nigeria, India. *Emerg Infect Dis, 21*(5), 908-909. doi:10.3201/eid2105.141804

Saksena, R., Matlani, M., Singh, V., Kumar, A., Anveshi, A., Kumar, D., & Gaind, R. (2017). Early treatment failure in concurrent dengue and mixed malaria species infection with suspected resistance to artemisinin combination therapy from a tertiary care center in Delhi: a case report. *Int Med Case Rep J, 10*, 289-294. doi:10.2147/IMCRJ.S139729

Santana, V. d. S., Lavezzo, L. C., Mondini, A., Terzian, A. C. B., Bronzoni, R. V. d. M., Rossit, A. R. B., . . . Nogueira, M. L. (2010). Concurrent dengue and malaria in the Amazon region. *Revista da Sociedade Brasileira de Medicina Tropical, 43*(5), 508-511.

Satyawali, V., Pandey, S., Rawat, V., & Khalil, M. (2014). Triple Co-infection of Malaria, Filaria, and Dengue: A Rare Entity. *J Lab Physicians, 6*(2), 136-137. doi:10.4103/0974-2727.141522

Serre, N., Franco, L., Sulleiro, E., Rubio, J. M., Zarzuela, F., Molero, F., & Tenorio, A. (2015). Concurrent Infection With Dengue Type 4 and Plasmodium falciparum Acquired in Haiti. *J Travel Med, 22*(5), 345-347. doi:10.1111/jtm.12222

Sow, A., Loucoubar, C., Diallo, D., Faye, O., Ndiaye, Y., Senghor, C. S., . . . Sall, A. A. (2016). Concurrent malaria and arbovirus infections in Kedougou, southeastern Senegal. *Malar J, 15*, 47. doi:10.1186/s12936-016-1100-5

Stoler, J., Delimini, R. K., Bonney, J. H., Oduro, A. R., Owusu-Agyei, S., Fobil, J. N., & Awandare, G. A. (2015). Evidence of recent dengue exposure among malaria parasite-positive children in three urban centers in Ghana. *Am J Trop Med Hyg, 92*(3), 497-500. doi:10.4269/ajtmh.14-0678

Suresh, V., Krishna, V., Raju, C., Teja, P., & Usha, V. (2013). A rare case of triple infection with dengue, malaria and typhoid. *Int J Res Dev Health, 1*(4), 200-203.

Swoboda, P., Fuehrer, H. P., Ley, B., Starzengruber, P., Ley-Thriemer, K., Jung, M., . . . Noedl, H. (2014). Evidence of a major reservoir of non-malarial febrile diseases in malaria-endemic regions of Bangladesh. *Am J Trop Med Hyg, 90*(2), 377-382. doi:10.4269/ajtmh.13-0487

Thaha, M., Pranawa, Yogiantoro, M., Tanimoto, M., & Tomino, Y. (2008). Acute renal failure in a patient with severe malaria and dengue shock syndrome. *Clin Nephrol, 70*(5), 427-430.

Thangaratham, P. S., Jeevan, M. K., Rajendran, R., Samuel, P. P., & Tyagi, B. K. (2006). Dual infection by dengue virus and Plasmodium vivax in Alappuzha District, Kerala, India. *Jpn J Infect Dis, 59*(3), 211-212.

Tulara, N. K. (2015). Concurrent infection with malaria, dengue and hepatitis A virus together. *Journal of Tropical Diseases & Public Health*.

Ward, D. I. (2006). A case of fatal Plasmodium falciparum malaria complicated by acute dengue fever in East Timor. *Am J Trop Med Hyg, 75*(1), 182-185.

Yasir, S., Rashid, M. O., Moin, F., & Owais, K. (2014). Co-existence of dengue fever & malaria in thrombocytopenic patients presented with acute febrile illness. *Pakistan Journal of Medicine and Dentistry, 3*(03), 19.

Yong, K. P., Tan, B. H., & Low, C. Y. (2012). Severe falciparum malaria with dengue coinfection complicated by rhabdomyolysis and acute kidney injury: an unusual case with myoglobinemia, myoglobinuria but normal serum creatine kinase. *BMC Infect Dis, 12*, 364. doi:10.1186/1471-2334-12-364

Yong, L. S., & Koh, K. C. (2013). A case of mixed infections in a patient presenting with acute febrile illness in the tropics. *Case Rep Infect Dis, 2013*, 562175. doi:10.1155/2013/562175
